# Advancing the Measurement of Personal Intelligence with the Test of Personal Intelligence, Version 5 (TOPI 5)

**DOI:** 10.3390/jintelligence7010004

**Published:** 2019-02-06

**Authors:** John D. Mayer, David R. Caruso, A. T. Panter

**Affiliations:** 1 Department of Psychology, University of New Hampshire, McConnell Hall, 15 Academic Way, Durham, NH 03824, USA; 2 Yale College Dean’s Office, Yale University, New Haven, CT 06510, USA; david.caruso@yale.edu; 3 L.L. Thurstone Psychometric Laboratory, Department of Psychology and Neuroscience, The University of North Carolina at Chapel Hill, Chapel Hill, NC 27599, USA; panter@unc.edu

**Keywords:** personality, personal intelligence, intelligence, measurement, factor analysis

## Abstract

People use their personal intelligence (PI) to understand personality in themselves and others. In Studies 1 and 2 (*N*s = 961 and 548), individuals completed the Test of Personal Intelligence, Version 5 (TOPI 5), which is introduced here. The TOPI 5 is an ability assessment with a broader range of content and more challenging items than earlier test versions. In past research, factor analyses indicated that people employ two distinct but highly correlated abilities to problem-solve in this area. These two-factor models, however, exhibited instabilities and limited applicability between the TOPI 4 and 5 in this research (and as reported in the Supplementary Materials). In Study 3, we successfully test the one-factor models of the TOPI with the present data and archival data sets (*N_archival_* = 19,627). We then use the one-factor models to develop a pair of new test forms: one that is compatible with all the TOPI test versions and another, TOPI 5E, that is better at distinguishing among people scoring in the higher range of performance relative to previous measures.

## 1. Introduction

Psychologists often view mental abilities as operating as a global intelligence, or *g*—a capacity to comprehend complex ideas, to reason, and to solve problems [1]. Contemporary measurement theory further divides *g* into a set of broad intelligences that include verbal-propositional, quantitative, and visuospatial intelligences, among others [2,3,4]. These broad intelligences correlate meaningfully with people’s performance in specific life areas, and these broad abilities can be used to increment predictions modestly above the use of *g* alone [5,6]. Present-day research also acknowledges the contribution of neuroanatomy to intelligence, as well as the contribution of culture to shaping the way intelligence is used and can be measured [7,8,9]. Experts have called for renewed studies of general intelligence and its subsidiary broad mental abilities [5,10,11]. 

Personal intelligence is a recently-proposed broad intelligence focused on the capacity to reason about one’s own and others’ personalities. More formally, it involves “the capacity to reason about personality and to use personality and personal information to enhance one’s thoughts, plans, and life experience” [12] (p. 209). To study the problem-solving involved, a *Test of Personal Intelligence* (TOPI) was introduced: an ability-based assessment consisting of multiple-choice items about personality that measures the accurate reasoning about personality traits, trait–behavior links, goals, and other aspects of understanding oneself and others [13].

TOPI scores correlate between *r* = 0.15 and 0.40 with other broad intelligences such as quantitative and verbal intelligences. The TOPI has a closer relation with ability-assessed emotional intelligence at *r* = 0.65 to 0.69, and so the two are sometimes referred to as examples of people-centered intelligences [14]. As with other intelligences, TOPI scores are mostly independent of measures of socio-affective styles and self-control, such as those measured by the Big Five—correlating *r* = 0.25 or less with Openness and Agreeableness and the rest [13,15,16]. 

The power of general intelligence (*g*) is little diminished by acknowledging that its subsidiary broad intelligences predict key outcomes beyond *g* alone [5,6,17]. Spatial and verbal intelligences predict criteria incrementally over general intelligence, accounting for an additional 2% to 6% of variance in performance level [6]. 

Personal intelligence, measured by the TOPI scales, also exhibits incremental validity over other indicators of intelligence and personality traits. Among other findings, mothers high in personal intelligence use more personality-related terms when speaking with their children, and their children use more personality-related terms as well, even after controlling for utterance length [18]. Students with higher personal intelligence are better able to fit their personalities to their college majors, controlling for (self-reported) SATs [19]. Moreover, personal intelligence incrementally predicted military cadets’ GPA in liberal arts courses (but not in the sciences), controlling for actual SAT scores, and predicted cadets’ reputations for good military performance [16]. People *lower* in personal intelligence experience greater conflict in their relationships and engage in counterproductive behaviors at work such as incivility, theft, and sabotage [20,21,22]. 

### 1.1. Advancing Measurement of Personal Intelligence 

#### The Test of Personal Intelligence (TOPI)—Content and Scoring

The TOPI has been developed over several versions, all of which employ multiple-choice items requiring people to solve problems relevant to personality. To ensure adequate content coverage, all full-length TOPI versions contain questions drawn from four theoretically-specified problem-solving areas: (a) identifying personality-relevant information; (b) forming accurate models of personality; (c) using personality-related information to guide choices; and (d) systematizing goal and actions [23,24]. For example, a question from the “forming accurate models” area asks: 

If a person is depressed and self-conscious, most likely, she also could be described as

- calm and even-tempered
- self-controlled
- anxious and impulsive
- fairly thick-skinned

Each test item has one correct answer (scored 1) and three distractors (scored 0). Correct answers are identified using a veridical scoring system keyed to specific research findings in the personality literature. The correct answer to the above item is “c. anxious and impulsive” because depression with self-consciousness describes a negative affect, which is associated closely with anxiety and impulsivity [25].

### 1.2. Key Foci of the Present Research

The present research is focused on two questions key to the development of valid tests: “How many mental abilities (i.e., factors) are involved in personal intelligence?” and “Is it possible to measure very high performance in the area better than has been the case in the past?”

#### 1.2.1. How Many Mental Abilities Make up Personal Intelligence? 

*Present status.* Evidence for a test’s validity comes in part from its factor structure—the degree to which the test and its subscales reflect empirically viable distinctions in attributes the test measures. In a recent article, we factor analyzed the TOPI 4 (earlier referred to as the TOPI 1.4)^1^ and found that personal intelligence could be divided into two “Consistency–Congruence” and “Dynamic–Analytic” personal intelligences. We then developed a TOPI 4-Revised (TOPI 4R) with subscales to assess them [15]. The TOPI 4 factor model, however, estimated the correlation between these two hypothesized abilities at *r* = 0.82 to 0.86, which was very high and suggestive of the alternative possibility that the TOPI measures just one ability. We concluded that “Although the two-factor approach fits well, an alternative one-factor model also was viable” [15] (pp. 308–309). 

*Problems with multiple factors in a broad intelligence.* Research by others near-contemporaneous with our 2017 project raised fresh challenges to the two-factor framework. First, a new review indicated that on average, broad intelligences already correlated among themselves at a high level of approximately *r* = 0.59 [21]. Schneider and Newman [6] estimated that broad intelligences, with their aforementioned average correlations of *r* = 0.59, added 2% to 6% of the variance of prediction over *g*. This leaves a relatively narrow range within which subfactors could correlate because they must fall sufficiently above that *r* = 0.59 level to be considered part of the *same* broad ability as opposed to a separate broad intelligence. At the same time, subsidiary factors within a given broad intelligence must be sufficiently distinct from one another to allow for incremental predictions. That leaves an effective target range of between, perhaps, *r* = 0.70 to 0.80—a challenging psychometric objective. When factors correlate so highly, it raises two further issues: their practical value and how stable and real they could be. Even if two factors correlated between *r* = 0.70 and 0.80 or so, the incremental value of one over the other is likely to be fairly limited, and that is what we have found so far with the two factors of the TOPI 4R [15]. Further, Legree, Psotka, Robbins, and colleagues [26] pointed out that artifacts, such as variations in the response scales across items, may create spurious factors (although all TOPI forms use the same response scales). 

Claiming that there are multiple related mental abilities when there is just one could lead to the creation of a test with spurious subscales—a problematic practice in assessment. As Sinharay, Puhan, and Haberman [27] (p. 29) note, “The common perception is that (a) subscores provide trustworthy information about the examinee’s strengths and weaknesses; and (b) the examinee will work harder on” areas of low scores so as to improve. But if that subscore information is inconsistent with the test’s actual factors, the scores can leave a test-taker misinformed—the opposite of a good testing practice. 

*The advantage of identifying multiple factors.* With these cautions concerning subfactors acknowledged, if the TOPI were constructed to measure one mental ability when personal intelligence was, in actuality, two or more abilities, that could lead to overlooking a test-taker’s genuine strengths and weaknesses by underrepresenting the skills involved. If there *really were* two or more mental abilities involved, those would be important to know about because (a) assessing them ensures that a person’s true strengths are acknowledged; (b) identifying distinct abilities can elucidate problem-solving strategies in the area and contribute to skills training; and (c) the finding could provide clues to the biopsychological systems in the brain that underlie intellectual functioning. 

#### 1.2.2. Improving the Score Distribution

The second focus of the present research was to improve measurement at the upper ranges of personal intelligence. The ability tests of people-centered intelligences (i.e., emotional, personal, and social) distinguish among performance levels below the 50th percentile effectively but perform less well in distinguishing people at higher levels of ability. 

For example, the *Situational Test of Emotional Understanding* is composed of items more sensitive to lower- than to upper-level emotional intelligence at a ratio of 3:1 [28], the Mayer, Salovey, Caruso Emotional Intelligence Test (MSCEIT) exhibits the same limitations [29] as do the TOPI 4th generation tests and its prior versions: Respondents who score below the 50th percentile exhibit a wide range of scores, but those who score above the 50th percentile are far less reliably distinguished. Put another way, the test score distributions in these instances are negatively skewed: more spread out among low-scorers and less distinguishable among the high. 

Perhaps these carefully-developed and valid tests all exhibit a negative skew because a natural limit exists to how clever people can be about understanding personality (or emotions) that places a cap on test-takers’ performance. Even if true, however, perhaps the tests could be improved somewhat in their upper registers by including more challenging test items; we attempted such improvements with the TOPI 5. 

### 1.3. The Research Approach 

#### 1.3.1. The Present Research

To keep measurement in the area on as solid a footing as possible, we revisited the issue of how many mental abilities make up personal intelligence and whether tests in the area could measure higher-than-average abilities with greater accuracy. To do this, we developed a more powerful test for this purpose, the TOPI 5. At 205 items and 13 problem-solving areas, the TOPI 5 was longer and included more diverse content relative to the 93-item TOPI 4th generation versions (the 4 and 4R); the test also was intended to be more challenging than the 4th-generation tests so as to assess higher ability levels.

As we will describe, the two-factor model we had developed earlier [15] failed to fit the Study 1 and 2 datasets collected here at the level we expected. At the outset of Study 3, therefore, we engaged in a series of clarifying, exploratory analyses, some on a trial-and-error basis, to help us better understand why we were seeing the results we had. (Guidance for obtaining the details of these analyses can be found in Section 1.3.2). The new analyses provided a more secure basis upon which to develop a factor model of the problem-solving—one that fit both the present data and archival data. 

#### 1.3.2. The Supplementary Materials

Please note that, in addition to the results reported here, we recorded additional details of our samples, methods, and procedures in an extensive Technical Supplement that appears both as the accompanying Supplementary Materials and as a document in an open-source online repository at the University of New Hampshire [30]; hereafter, we will refer chiefly to the repository document [30]. The online locations are indicated in the section on Supplementary Materials at the end of the article, and in the reference referring to the Technical Supplement [30].

## 2. Study 1

*Hypotheses.* In Study 1 we addressed whether (a) the two-factor TOPI 4 structural model (Model 4) that we developed using the TOPI 4 tests would generalize to the longer, more diverse TOPI 5; and (b) whether a one-factor model also would fit sufficiently well to indicate its viability. Note that “Model 4” refers to the two-factor model of the TOPI 4 and 4R; no Models 1, 2, or 3 were proposed.

### 2.1. Method

#### 2.1.1. Participants

Participants were 961 students taking psychology courses at a large New England public university who participated in a human subject pool and who met the criteria for completeness of responses and reasonable levels of attention. To obtain a desirable sample size, the survey was available to students in the subject pool in exchange for a course credit for most semesters between Fall 2014 and Spring 2017. 

Of the 1310 who had logged onto the survey, 69 were non-respondents (completing just a few items) and 94 were partial respondents (completing less than 50% of the TOPI survey), and these were removed, leaving 1147 in the sample. An additional series of screens flagged and removed participants for signs of extreme inattention [30] (Chapter 3), yielding a final sample of *N* = 961. The final sample included 670 women and 288 men (10 unspecified) with a *M_age_* = 19.5 years, of which 867 self-identified as White, followed by smaller groups who self-identified as Asian or African-American, and then smaller numbers of other groups [30] (see Table 6.1).

#### 2.1.2. Measures

*Demographics.* Participants were asked their age, gender, and ethnic/racial identification. 

*The Test of Personal Intelligence, Version 5 (TOPI 5)*. The TOPI 5 is a 205-item multiple choice test of personal intelligence with items of the type described in the introduction. Version 5 included nine tasks making up the TOPI 4th generation tests (reflecting the merger of two pairs of the original tasks from the TOPI 4R, based on their similarity) and four new tasks including measures of defense mechanisms and personality change developed over item research studies in 2013 and 2014, with *Ns* of 446 and 381 [30] (see Chapter 2). All new tasks included items intended to be more difficult so as to better measure higher ability levels relative to prior versions of the test. The tasks and brief descriptions are indicated in Table 1. The test also included multiple-choice attention-check items dispersed in small subsets of one, two, or three throughout the test. 

#### 2.1.3. Procedure

Participants logged onto the participant–management system, SONA, and were transferred to an online survey hosted by Qualtrics. Given the survey length and its cognitive demands, we suggested participants take a few-minute break sometime during the testing. To control somewhat for fatigue effects, the TOPI 5 was presented online in two forms, the original order and a “second-part-first” order, in which the last seven tasks were moved to the beginning, and the first six tasks were moved to the end. All items were presented in a fixed order, other than that counterbalancing. The two orders can only address the effects of fatigue in part so, as noted, we also screened participants for attention. 

### 2.2. Study 1 Results

#### 2.2.1. Analyses Reported in this Article and in the Accompanying Supplementary Materials

We focus our report of analyses on the factor analyses of the TOPI 5’s 205 items. We also conducted analyses at the level of the test’s 13 tasks, leading to similar conclusions as those drawn at the item level [30] (see Chapter 5 for details).

#### 2.2.2. General Approach to Factor Analyses 

*Treatment of data and related technical specifications.* In our Mplus analyses [31], we treated the item-level data as categorical and employed the Weighted Least Squares Mean and Variance adjusted estimation (WLSMV) that is especially suited to such data. We obtained oblique factors employing a facparsim algorithm, owing to its often-superior handling of large numbers of variables. 

*Fit criteria.* We set the conventional fit criteria for our factor models “close to” 0.95 or higher for the Bentler Comparative Fit Index (CFI) and Tucker–Lewis Index (TLI) and “close to” 0.06 or lower for the Root Mean Square Error of Approximation (RMSEA) [32]. We added the further criterion that factors should correlate near *r* = 0.90 or below, as values above 0.90 can imply that the two factors are approximately the same. 

*The 58 common items.* To ensure our models were consistent across test forms, we identified a set of 58 items that the TOPI 4R and TOPI 5 shared and that met the criteria for functional items in the present sample (all items loaded >0.35 on their primary factor and no higher than *r* > 0.25 on their secondary factor; 4 common items of an original 62 were excluded). Fortunately, the 58 items were about evenly divided between the two TOPI 4R factor scales of Consistency–Congruency and Dynamic–Analytic. 

*Fit of the (58-item) Model 4 to the original TOPI 4R data.* The 58-item version of Model 4 fit the original data (from “Archive A” of [15]) on which the TOPI 4R was developed even better than the full 67-item Model 4 had, with an RMSEA of 0.013 for the 58 items and an excellent fit of CFI and TLI of 0.957 and 0.956 with an interfactor *r* = 0.82. 

#### 2.2.3. Did a Two-Factor Model 4 Fit the TOPI 5 data? (Hypothesis a)

We conducted an initial test of the two-factor model with the Study 1 data by examining the results from an exploratory factor analysis of the test (Table 2). The two-factor exploratory model fit with an RMSEA of 0.008 and with CFI and TLI both of 0.984 with a *r* = 0.40 correlation between the factors—an excellent result.

Although the two-factor exploratory model fit the TOPI 5 data quite well, there was no assurance those were the *same* two factors as reported in our 2017 report. To test Hypothesis *a* more exactly, we applied Model 4 to the TOPI 5 data in a confirmatory factor analysis. Despite the fine retrofit of the 58-item version of Model 4 using the earlier TOPI 4 data, the same model failed to provide a reasonable fit to the present data: its RMSEA was good at 0.037, but its CFI and TLIs were 0.882 and 0.877, with an estimated correlation between the factors of *r* = 0.92. (These values, along with further analyses, also are reported elsewhere [30] (see Chapter 6, Table 6.2, under the TOPI 4 Model heading, in the Study 1 row).

We noted as well that the one-factor exploratory model fit far better than the two-factor Model 4. 

In plain terms, these results suggest that our 2017 TOPI 4 Model (Model 4) was robust for the TOPI 4 but failed to generalize to the TOPI 5 for which another model appeared necessary. These findings were unexpected, so we sought to learn more about them going forward.

#### 2.2.4. Post-Hoc Hypothesis: Might a Two-Factor Model Constructed from the TOPI 5 Data Itself Better Fit the Data? 

We further wondered whether a two-factor model based on the TOPI 5 data collected here—a “Model 5” (where the “5” is for the TOPI 5) might provide an alternative that fit the present data better—as well as fit other data sets we had collected earlier.

*Developing a two-factor TOPI 5 model.* Working from the two-factor exploratory factor analysis for the TOPI 5, we re-divided the 58 items depending upon whether their highest loading was on Factor 1 or 2 in the present sample, using the exploratory factor analysis as a guide. The item placements were relatively straightforward because 51 of the 58 items possessed loadings were >0.20 higher on one factor than the other. By coincidence, the TOPI 5 model had 27 items on Factor 1 and 31 on Factor 2, the same numerical split as the TOPI 4 model but with some items changing factors. 

The TOPI 5 model fit the TOPI 5 sample quite well with an RMSEA of 0.016 and CFI and TLIs of 0.977 and 0.976, respectively, and the correlation was *r* = 0.61, indicating a good deal of independence and reflecting a viable overall fit for a two-factor model of mental ability in the area. The 58-item TOPI 5 model failed to fit the data collected earlier with the TOPI 4, however [30] (Chapter 6). 

*Another fly in the ointment.* We found the two factors of the TOPI 5 nearly impossible to interpret. We note that the two-factor model of the TOPI 4 also had been challenging to interpret but the two factors here seemed to defy description altogether, despite our collective experience at interpreting factors [30] (Chapters 3 and 5). 

#### 2.2.5. To What Degree Does the Factor Structure Support the Idea of Personal Intelligence as a Single, Broad Mental Ability? (Hypothesis b) 

Hypothesis *b* in Study 1 was that the factor solutions would support the view of personal intelligence as a single, unitary ability, regardless of their support for two subsidiary abilities. The initial support for a one-factor model came from the evidence that the TOPI 4 model estimated the factors of the two-factor model correlated at *r* = 0.92, indicating a high degree of similarity between them. In fact, a one-factor model of personal intelligence fit the TOPI 5’s 205 items rather well in the exploratory factor analysis, with an RMSEA of 0.015 and with CFI and TLI both of 0.940. The reasonably good fit occurred absent any initial screening for problematic items. This finding argues, in effect, that personal intelligence encompasses a very broad number of problem-solving areas about personality—not only those we have studied before but also the newly-added problem-solving domains that concerned mental defenses, trait-to-behavior relations, and personality change. Collectively the results were supportive of the single broad ability view of the problem solving. 

### 2.3. Study 1 Discussion

In Study 1, our attempt to validate the two-factor TOPI 4 model of personal intelligence on data from the TOPI 5 was disappointing: The TOPI 4 model failed to fit the TOPI 5 data. This, in turn motivated the development of a new TOPI 5 model, “Model 5”, that did fit the TOPI 5 data but not the TOPI 4. 

Two bright spots were that (a) we successfully fit one- and two-factor models to the TOPI 5; and (b) the model results argued clearly for the overall cohesive quality of this broad ability, both because a one-factor model fit the data reasonably well and because the two-factor TOPI 5 model estimated a high correlation between the factors. These results enlarge our conception of personal intelligence because the TOPI 5 test included new problem-solving areas relative to the TOPI 4 including (a) recognizing and explaining mental defenses; (b) trait-to-behavior relations; and (c) personality change. 

We came up with two possible explanations for the failure of the TOPI 4 model to generalize to the TOPI 5 data: (a) we had misidentified some of the 58 items that were common to the TOPI 4/4R and 5 tests; and (b) the discrepancies between the findings for the two-factor models were due to some unknown, anomalous behavior of our most recent sample. Regarding explanation “a”; we triple checked the items and found no errors. To test explanation “b” (e.g., something anomalous about this sample), we attempted to replicate our findings in a new sample in Study 2. 

## 3. Study 2: Attempt to Replicate Study 1 

We attempted to replicate the findings of Study 1 in Study 2 using the TOPI 5R, a 145-item abridged version of the TOPI 5. Our central hypotheses were that (a) the two-factor TOPI 4 model would *not* fit the sample data, (b) the TOPI Model 5 would fit the here-abridged TOPI 5, and (c) that a one-factor model also would fit the new data. 

### 3.1. Method and Procedures

Study 2 was parallel to Study 1 in almost all respects: Participants were drawn from the same population and from the same subject pool, tested online the same way, awarded the same credits, and screened identically. All data were collected in the Fall 2017 semester. Of the *N* = 686 initial logins, *N* = 548 remained after removing non- and partial-respondents and those flagged for extreme inattention. All subsequent analyses were conducted on this final sample, which included 371 women and 175 men with a *M_age_* = 19.0 years, of which 506 self-identified as White, followed by a smaller group of participants self-identifying as Hispanic/Latino, and then other groups [30] (see Chapter 6, Table 6.1 for details).

The TOPI 5R was abridged from the TOPI 5 to 145 items first by removing 55 items that failed to load >0.35 on either factor of the two-factors in the Study 1 two-factor exploratory factor model and second by removing items that correlated >0.25 on its secondary factor; we further removed the personality change and act frequencies tasks in their entireties after the item screening left so few items that the tasks lacked reliability. Due to the TOPI 5R’s shorter length of 145-items, we presented it to the respondents in one fixed order, followed by several additional scales that are not further considered here [30] (Chapter 4). 

### 3.2. Study 2 Results

Using the newly-collected data, we tested our three hypotheses.

#### 3.2.1. Did the two-factor TOPI 4 model fit the TOPI 5R data of Study 2 (Hypothesis a)? 

A confirmatory factor analysis of the two-factor TOPI 4R model again did not support its fit, with a reasonable RMSEA = 0.029, a somewhat lower CFI and TLI = 0.904 and 0.901 respectively, and an untenable relation between the factors of *r* = 0.94 [30] (see Chapter 6, Table 6.2, left side, Study 2 row).

#### 3.2.2. Did the two-factor TOPI 5 model fit the TOPI 5R data of Study 2 (Hypothesis c)? 

A further confirmatory test of the two-factor TOPI 5 model did fit the data about as well as on the initial TOPI 5 data set. More specifically, the model yielded an RMSEA = 0.017, and CFI, TLI = 0.969 and 0.968 respectively, and an *r* between factors of *r* = 0.62: an overall excellent picture [30] (see Table 6.2, Study 2 row, right side bottom). 

### 3.3. Study 2 Discussion 

In Study 2, the two-factor TOPI 4 model again failed to fit the data, and the two-factor TOPI 5 model again did fit the TOPI 5. The inconsistent performance of the two-factor models across forms found in Study 1 appeared real rather than anomalous.

## 4. Study 3: The Application of One-Factor Models Across Six Data Sets and the Development of New TOPI Versions on That Basis

In Study 3, we hoped to develop new one-factor models of the TOPI that more reliably fit across the data sets in our possession and to develop TOPI versions based on those models. To ensure the viability and generalizability of the new one-factor test models, we assembled four archival data sets on which to check the models and continued to employ the data samples collected in Studies 1 and 2. A second aim was to see if we could use new items from the TOPI 5 to better measure the higher realms of ability than in the past.

### 4.1. Archives A, B, C, and D 

The four data archives contained predominantly college-age students working toward their baccalaureates, with some attending United States military academies and/or enrolled in the United States Reserve Officer Training Corps (ROTC), and others with civilian status. Archive A was composed (95%) of college students enrolled in military academies and ROTC and Archive B was of military academy and ROTC students as well (*N* = 8459). Archives A and B were described in [15], where they were referred as the Original and Replication samples, respectively. We constructed Archive C (*N* = 4922) for this study from datasets from the same military population of test respondents, whose responses were sent to us between July 2015 and June 2018 from the *Office of Economic and Manpower Analysis* of the United States Army (OEMA) for scoring. These data were scored shortly after they were sent but were otherwise left unanalyzed by our research group until this project.

Archive D was from a predominantly civilian college student group (*N* = 1072) who participated in graduate-student research projects and whose de-identified data was available to us. The sample was drawn from a large Northeastern campus. A few non-civilian students also may have been among the group as the campus of approximately 12,000 students hosted “over 100” ROTC members at the time [33]. 

The average age across the groups varied from 18.5 to 21.1 years. The three military archives A, B, and C had more men than women (about 3.5 to 1), but women predominated in the civilian samples (about 2.5 to 1). The estimated ethnic compositions of the military and civilian samples were 78% and 91% White/Caucasian, respectively, with the remaining groups from Black/African, Asian, Hispanic/Latino groups next, and then the representations from other groups as well [30] (see Table 6.1 for details).

### 4.2. A Note on Further Analyses of Clarification

Before proceeding with the one-factor models, we felt a considerable responsibility to understand how the earlier two-factor TOPI 4 model, which had been developed using data from large sample (*N* = 5144), cross checked on a holdout group (*N* = 5174) and replicated in an independently-collected sample (*N* = 8459) had nonetheless failed to generalize to the new scale and samples. 

We therefore tested several hypotheses using the six datasets we had assembled, including exploratory hypotheses and further tests of a trial-and-error nature. These analyses are reported midway through Chapter 5 and further in Chapter 6 of the Technical Supplement [30]. 

To summarize our results here, we found no effect dependent upon the composition of the samples we collected (some samples included those in the military; other samples were civilian test-takers). The difference in the model fit instead appeared dependent on the specific version of the TOPI in which our 58 common items were embedded. When the 58 items were embedded in the TOPI 4th generation tests, the two-factor TOPI 4 model fit best. When the same 58 items were embedded in the TOPI 5th generation tests, however, the two-factor TOPI 5 model fit best. In other words, the two-factor models most likely reflected artifacts due to item-context effects rather than variance reflecting two truly distinct mental abilities. These exploratory analyses argued strongly for the one-factor approach with which we continued in Study 3.

### 4.3. Study 3: New One-Factor Scales? 

*Research Approach.* To examine the possibility that one-factor TOPI models might fit more consistently across data sets and would thereby provide a more secure foundation for scale scores moving forward, we attempted to fit the one-factor TOPI 4 and TOPI 5 models to the six sets of data.

*Hypotheses.* We tested two hypotheses: (a) that we could create a one-factor model and measures drawn from the 58 items common to the TOPI 4th and 5th generation tests that would fit across all six data sets examined here and (b) that the new items on the TOPI 5 would do a better job of measuring the upper reaches of personal intelligence than the earlier TOPI 4 items and that, by using selected TOPI 5 items, we might develop a personal intelligence measure that better assessed higher levels of ability than in the past. 

### 4.4. Method

#### Samples and Procedure 

We fit our models across all six datasets employed here to test our hypotheses. Elsewhere, we report the invariance analyses consistent with the findings here [30] (Tables 6.4 and 6.5).

### 4.5. Results

#### 4.5.1. Could a One-Factor Model of the 58 Items Common Across Data Sets Fit with Consistency? (Hypothesis a)

We applied a one-factor model to the 58 common test items across the six datasets, separately on each set. The resulting six sets of fit statistics are indicated in the upper portion of Table 3. The one-factor model fit Archives A through D well (the TOPI 4 and 4R data), but failed to meet the criteria for the Study 1 data and was borderline in the Study 2 data (TOPI 5 and 5R). 

*Refining the one-factor model.* We next examined the model’s modification indices (MIs) for the Study 1 dataset, where the model especially ran into difficulty. (We also examined the MIs in Archive A—a very differently behaving data set—to identify any consistencies between the Archive A and Sample 1 data sets.) The MIs, which estimate the change in χ² that result from freeing the fixed parameters, indicated that a small number of item pairs violated the one-factor model in Study 1 (as well as in the earlier data). We identified and removed 11 items that, in Study 1, either had appeared in a pair with a relatively high modification index or that appeared repeatedly in problematic pairs. The result was a 47-item TOPI version that we referred to, hopefully, as *Version 5 General* (TOPI 5G), where the “general” denoted both a single general factor and (we hoped) the test’s good generalizability across multiple forms of the test and data sets. The TOPI 5G, with its reduced 47 items, exhibited good-to-excellent fit across all six datasets (see Table 3, middle-lower rows) and, in every data set, outperformed the original one-factor model fits that had used all 58 common items. 

#### 4.5.2. Could a Scale that Employed the New TOPI 5 Test Items Better Measure the Upper Reaches of Personal Intelligence? (Hypothesis b) 

*An item response theory (IRT) approach to item difficulty.* To determine whether using new items from the TOPI 5 could lead to a better measurement at higher ability levels of personal intelligence, we created a 66-item TOPI 5E (*E*xtended measurement) that included more challenging items, as indicated by the *b* parameter from a 2-parameter IRT model. The IRT model estimates *a* and *b* parameters for each item, where *a* indicates the item’s capacity to discriminate the ability being measured (i.e., personal intelligence) and *b* indicates the level-of-ability at which the item best discriminates among test-takers (similar to item difficulty). The approach was applied to the 170 items of the TOPI 5 that loaded >0.35 on its first factor, concluding with the 66-item form 5E.

A one-factor confirmatory factor analysis performed on the TOPI 5E items fit the new test reasonably well across the Study 1 and 2 samples (Table 3, bottom rows). Note that only 56 of 66 items of the 5E had been carried forward to the TOPI 5R and could be tested using the Study 2 data. We referred to that abbreviated scale as the TOPI 5RE. 

The descriptive statistics for the TOPI 5G and 5E (and the 5RE, the abbreviated 5E used in Study 2) are indicated in Table 4. Both the 5G and 5E exhibited excellent coefficient alpha reliabilities of between *r* = 0.89 to 0.93 and IRT marginal reliabilities of *r* = 0.86 to 0.91. The TOPI’s negative skew was reduced from the TOPI 5G to the 5E from −1.01 to −0.79, *t* = 2.78 and *p* < 0.001. (We do not further recommend the use of the 5RE as it excludes several excellent items, and so do not further consider it.)

**Figure 1:**
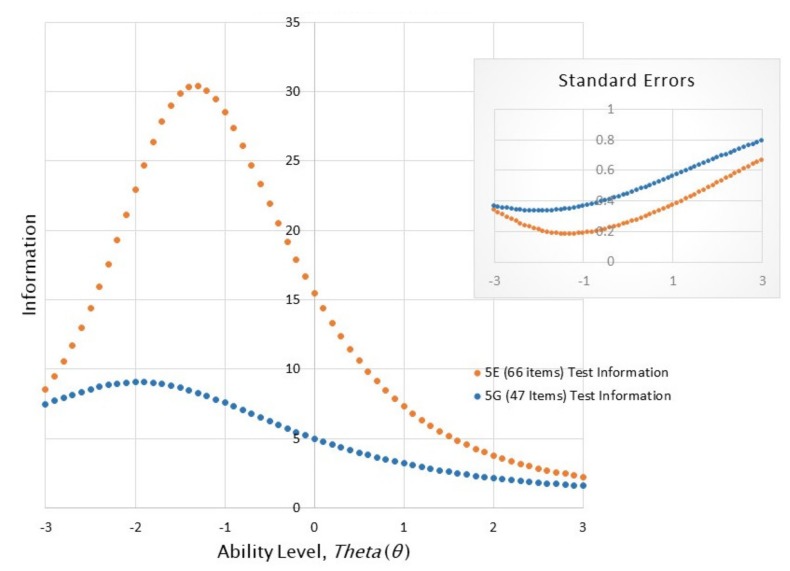
The test information curves and standard errors for the TOPI 5E and 5G.

Figure 1 indicates the total test information curves for the TOPI 5G and 5E on the Study 1 sample of *N* = 961. The *x*-axis indicates the participants’ ability level (theta) in standard deviations around the mean. The *y*-axis indicates the information the test returns at a given level of test-taker’s ability. The test information is calculated in part from the reciprocal of the test’s conditional standard error: When the curve is higher, the test returns more information. The test standard error curves also are depicted in the upper right insert. The information curve for the TOPI 5E is much higher than that for the 5G indicating its greater overall informativeness. Most importantly, the test is more informative (i.e., the curve is higher) between one- and two-standard deviations above the mean performance level relative to the TOPI 5G. Although the TOPI 5E remains less informative than might be desirable at its high reaches, it represents a substantial improvement over the 5G in returning both more information on the whole and consistently smaller standard errors.

### 4.6. Further Examination of the TOPI 5G AND 5E

*Equivalency of validity among one-factor scales and between one- and two-factor models.* Some of the correlations between the test forms and the common set of 58 items are indicated in the last column of Table 4. Adjusting those correlations for attenuation due to unreliability, we found that the estimated correlation among the true scores were essentially one: For the TOPI 5G and 5E forms with the TOPI 4R (using just the 58 common items), the values were *r*_4__w5G_ = 1.08 and *r*_4__w5E_ = 1.01, and for the *r*_5G__w5E_ = 1.01 (values above 1.0 are an artifact of the shared items). The tests measure exactly the same attribute.

*Correlations of the TOPI 5G with criteria.* Evidence for a test form’s validity comes from its correlation with criteria. To double-check the validity of the TOPI 5G, we reanalyzed the data from an earlier article concerning employees with high- and low-personal intelligence [22], calculating the TOPI 5G scores using the original item-level data from the TOPI 4R. The 5G’s predictions for several key criteria were approximately the same as that for the 4R, typically midway between the two factor-based scales, and in two instances, slightly above their predictions. Overall, the TOPI 5G predicted outcomes from *r* = 0.00 to 0.03 lower than the correlational value for the two factor-based scales averaged together (for example, *r* = −0.22 versus −0.24), a difference that can be accounted for nearly in full by the shorter length and consequent slight reduction in reliability of the TOPI 5G relative to the TOPI 4R (α = 0.90 versus 0.92 in this sample). The correlations with the criteria are consistent with those obtained in the field more generally [34] (for example). Regarding the questions of incremental validity, predictions from the TOPI occurred whilst a measure of vocabulary included for comparison purposes failed to exhibit similarly strong relations with the criteria. In practice, the relative brevity of the TOPI 5G could encourage participants to answer it more carefully and yield correlations that are equivalent or better than those of the TOPI 4R. 

## 5. General Discussion

### 5.1. Summary of Findings

Models of human intelligence are key to understanding a person’s intellectual strengths and weaknesses. Measuring more intelligences than exist can misrepresent a person’s mental capacities; measuring too few can overlook an individual’s capabilities. In our 2017 article [15], we reported a measurement model for the *Test of Personal Intelligence, Versions 4 and 4R* that represented the mental ability as two correlated but distinguishable mental abilities (*r* = 0.82 to 0.86). 

One key reason we developed the TOPI 5 introduced here was to further clarify our understanding of how many distinct mental abilities made up personal intelligence. We believed that a longer test (205 vs. 93 items), drawn from a wider group of problem-solving areas (13 vs. 9), would provide a more definitive model of people’s mental abilities. The present reexamination of the earlier two-factor TOPI 4 Model revealed a limit to its generalizability. The two-factor TOPI 4 Model replicated well in two newly constructed archives of TOPI 4 test-takers [30], but failed to generalize to samples taking the TOPI 5 and 5R tests (Studies 1 and 2 here). Although the two-factor Model 4 may be valid for the TOPI 4, the evidence here suggested that Model 4 may have fit well due to the context effects from test’s specific content. We also found two (different) factors in the TOPI 5 that failed to fit the TOPI 4 data sets. The findings strongly supported one-factor models as better in generalizing well across 4th and 5th generations of the test. See also [30] (Chapters 6 and 10), for further rationales for this change.

In Study 3 of this paper, we introduced what we hoped will be more robust, one-factor forms of the TOPI tests: a TOPI 5G that was backward-compatible with versions 2 through 5 of the test and the TOPI 5E that, although not fully compatible with earlier versions, better assessed higher levels of personal intelligence than the TOPI 5G. 

### 5.2. Advantages of a One-Factor Approach

Perhaps the chief advantage of the one-factor approach to personal intelligence is that the one-factor model fits multiple samples and forms of the TOPI test. Until such a time as stable subfactors are identified, reporting a single score that reflects a test-taker’s personal intelligence makes sense. 

A one-factor model of personal intelligence also is arguably more compatible with contemporary models of intelligence, in which other broad intelligences are regarded as single factors, represented by individual tasks, and lack further division into subfactors. An additional advantage is that there is no longer a need to remove items that load on two factors; that permitted us to retain a wider selection of test items on the TOPI 5E (extended version), and some of those items, it turned out, better discriminated among people higher in personal intelligence than items on prior tests.

### 5.3. Concluding Comments

*Limitations.* After careful consideration of our empirical findings, we concluded that despite their good fit to certain data sets (but not others), two-factor models of personal intelligence do not reflect true mental qualities, but rather most likely reflect test artifacts. We therefore argue for a one-factor approach to tests in the area. The weight of the evidence at present is that personal intelligence is best considered unitary.

This discussion of factor structure also may bring to mind the challenging question of how personal and emotional intelligences relate because they, too, overlap considerably, with personal intelligence correlating about *r* = 0.69 with strategic emotional intelligence (the latter includes emotion understanding and management, which arguably may be closest to personal intelligence in nature [13]. The correlation between personal intelligence and *overall* emotional intelligence remains unknown. 

*Recommendations.* Researchers may wish to continue using the overall TOPI 4R overall score in their research (the TOPI 4 overall score averages the two earlier-distinguished factors), as it provides an excellent representation of the test-takers’ abilities. Alternatively, they can rescore the test using the TOPI 5G, which conforms more directly to the one general factor model of the test. We estimate the overall TOPI 4R and TOPI 5G scores correlate about *r* = 1.0 once corrected for test unreliability, so it makes little difference which is employed. We suggest, however, deemphasizing the interpretation of the two factors of the TOPI 4R, as they seem more likely to have emerged from the test context rather than to reflect truly distinct mental functions. We further recommend the use of the TOPI 5E owing to its possibly greater precision in identifying the participants who perform at higher ability levels. 

In relation to training and education, the theory of personal intelligence distinguishes between test content (for example, areas of forming accurate models of personality and of systematizing plans), on the one hand, and the structure of abilities people bring to solve such problems, on the other. The theory can be used in education to divide the coverage of personal intelligence into content areas for the purposes of teaching. But although teaching might well proceed by content area to help organize the instruction, personal intelligence itself seems to be best represented as a global reasoning rather than distinguishing more specific ability areas. 

It *is* worth pointing out, however, that although a person may have a single ability in the area, their knowledge of specific content areas about personality may be helpful to assess in the form of educational quizzes or tests that assess the knowledge of one area or another. Our own testing experience does not preclude the possibility of such measures.

*Personal intelligence matters.* Whether or not personal intelligence ends up being best described as one mental ability or as two highly related ones, the TOPI test scores appear to represent a broad intelligence in that they correlate *r* = 0.15 to 0.40 with other commonly studied broad intelligences (and *r* = 0.65 to 0.69 with ability-measured emotional intelligence). 

The issues examined here regarding the test factor structure and uneven measurement precision across the ability levels should not obscure the promise already exhibited by the TOPI tests in moderately incrementing the predictions of consequential outcomes after controlling for other mental abilities such as verbal and quantitative intelligences (and utterance length in developmental studies) and after controlling for broad socioemotional traits such as those found in the Big Five. 

The predictions from the TOPI include that students with higher PI exhibit a superior performance in liberal arts courses, choose college majors they enjoy more, and are more likely to stick with those majors; it includes that employees with higher PI engage in less counterproductive behavior on the job, and that members of couples with higher PI exhibit less conflict in relationships, among other findings [16,19,20,22]. 

Looking toward the future, we hope that the present studies will provide a more secure foundation for new research in the area. We clarified that one overall score may be sufficient to capture the reasoning ability in the area and that it is possible to improve the assessment of test-takers who have high skill levels in the area, although there appear to be natural limits as to how clever one can be in people-centered reasoning. 

## Figures and Tables

**Table 1:** An overview and reference guide to TOPI clusters by area in the Test of Personal Intelligence Versions 2 through 5.

Abbreviated Task Name	Brief Task Description	
	Given	Solve for
	*Identifying Personality-Relevant Information (RVx Tasks)*	
Identifying Motives	● several behaviors and/or pursuits	● the common motive among them
Inner States	● a situation, activity, or role in which a person is engaged	● infer a person’s inner state from information
Evidence about the Self	● a need for information about oneself	● ways to receive accurate feedback
Inner Experience-to-Behavior	● a person is carrying out a common activity	● identify an inner experience that likely accompanies that activity
	*Forming Models of Personality (FMx Tasks)*	
Trait Knowledge	● a person possesses two traits	● the person’s third likely trait
Integrating Information	● several personality-relevant pieces of information	● a characteristic of the person’s knowledge, intellect, or beliefs
Discrepancies-Defense	● a discrepancy between a person’s words and behavior	● infer something about a person’s defense and coping
Act Frequencies	● a person’s trait	● behaviors associated with it
	*Guiding Choices Using Personality-Relevant Information (GCx Tasks)*	
Trait Inferences	● someone’s trait(s)	● the person’s likely reaction in a situation
Observers’ Trait Ascriptions	● an observer’s plans or behaviors around a target individual	● identify the trait that an observer ascribes to the target person
Motivating Memories	● a person’s motivational need	● identify the personal memory that will enhance the individual’s motivation
	*Systematizing Plans and Goals (SGx Tasks)*	
Goal-Related Subsidiary Actions	● a longer-term goal	● an intermediate or subsidiary goal, attitude or behavior that could satisfy it
Goal Evaluation	● a person’s objective (e.g., to make friends)	● a goal that likely will create conflicts for the person because it is unrealistic, hard to fulfil, or contradicts the aim
Personality Change	● a person’s intentions and behaviors	● how ready they are to change
	*Discontinued Tasks*	
Room with a Cue	● a person’s physical surrounding	● infer some relevant traits
Trait Judgeability	● several traits	● which are most visible/judgeable
Misc. Hard TOPI Questions	—	—

**Table 2:** The Exploratory Factor Analyses at the item level for Studies 1 and 2.

***Study 1*** **Item-Level Exploratory Factor Models of the TOPI 5 (*N* = 961) for 205 Items ^a^**							
**No. of Factors**	**Dep. Vars./Free Params.**	**Fit Indices**					***r*s among factors**
		Chi-2	df	RMSEA	CFI	TLI	*min to max*
One	205/205	25135.28	20705	0.015	0.940	0.940	NA
Two	205/409	21673.45	20501	0.008	0.984	0.984	0.40
Three	205/612	21143.92	20298	0.007	0.989	0.988	0.23 to 0.47
Four ^a^	NA	NA	NA	NA	NA	NA	NA
Five	205/1015	20477.52	19895	0.006	0.992	0.992	0.15 to 0.51
***Study 2*** **Item-Level Exploratory Factor Models of the TOPI 5R (*N* = 548) for 145 Items ^a^**							
**No. of Factors**	**Dep. Vars./Free Params.**	**Fit Indices**					***r*s among factors**
		Chi-2	df	RMSEA	CFI	TLI	*min to max*
One	145/145	11759.49	10295	0.016	0.951	0.950	NA
Two	145/289	10579.86	10151	0.009	0.986	0.985	0.40
Three	145/432	10336.32	10008	0.008	0.989	0.988	0.26 to 0.54
Four ^a^	145/574	10147.44	9866	0.007	0.991	0.990	0.24 to 0.52
Five	145/715	9975.38	9725	0.007	0.992	0.991	0.22 to 0.47

^a^ The four-factor solution for the TOPI 5 did not converge.

**Table 3:** The Study 3 model fits of the factor analyses ^a^ of the one-factor models of the TOPI 5G and 5E across relevant archives and samples.

Archive and Source	*N*	Items/Item Splits	Variables/Free Parameters	Chi-2	df	RMSEA	CFI	TLI
TOPI One-Factor Exploratory Factor Analysis for the 58 Common Items								
*Archives of Military and Civilian Test-Takers on the TOPI 4 and 4R*								
A ^b^, military	5174	58	58/116	3565.35	1595	0.015	0.939	0.937
B, military	8459	58	58/116	5506.22	1595	0.017	0.946	0.944
C, military	4922	58	58/116	4143	1595	0.018	0.945	0.943
D, civilian	1072	58	58/116	2248.73	1595	0.020	0.938	0.936
*Study 1 and 2 Samples Taking the TOPI 5 and 5R*								
TOPI 5 Sample	961	58	58/116	3687.62	1595	0.037	0.879	0.874
TOPI 5R Sample	548	58	58/116	2355.40	1595	0.029	0.903	0.900
TOPI-5G Confirmatory Factor Analyses								
*Archives of Military and Civilian Test-Takers Taking the TOPI 4 and 4R*								
A ^b^, military	5174	47	47/94	2034.57	1034	0.014	0.962	0.960
B, military	8459	47	47/94	3096.99	1034	0.015	0.965	0.963
C, military	4922	47	47/94	2308.46	1034	0.016	0.965	0.964
D, civilian	1072	47	47/94	1397.20	1034	0.018	0.960	0.958
*Study 1 and 2 Samples Taking the TOPI 5 and 5R*								
TOPI 5 Sample	961	47	47/94	1828.27	1034	0.028	0.943	0.941
TOPI 5R Sample	548	47	47/94	1491.87	1034	0.028	0.939	0.937
TOPI 5E Confirmatory Factor Analyses								
TOPI 5 Sample	961	66	66/132	4086.01	2079	0.032	0.940	0.938
TOPI 5R Sample	548	56	56/112	2085.00	1484	0.027	0.952	0.951

^a^ Note that for 1-factor models only, the exploratory and confirmatory factor analysis fit statistics are the same. ^b^ The even half, a.k.a., the cross-check sample of Archive A is reported here for purposes of direct comparison with the statistics reported in Study 1 of [15] (the odd-numbered participants’ data was used for the model construction).

**Table 4:** The characteristics of the TOPI 4 58 items common to Model 4 and 5, the TOPI 5G, and TOPI 5E and 5RE in Studies 1 and 2.

Test Form or Item Group	Items	M	S	Skew	S.E. Skew	Alpha Reliability	Marginal Reliab., IRT	RMSEA for IRT Fit	*r* with *M*_level_ on the 58 Common Items
	Study 1 Sample *N* = 961								
58 common items	58	0.763	0.171	−1.15	0.079	0.92	—	—	1.00
TOPI 5G	47	0.738	0.181	−1.01	0.079	0.90	0.86	0.03	0.98
TOPI 5E	66	0.700	0.188	−0.79	0.079	0.93	0.91	0.05 ^b^	0.93
	Study 2 Sample *N* = 548								
58 common items	58	0.735	0.165	−0.86	0.104	0.90	—	—	1.00
TOPI 5G	47	0.706	0.179	−0.71	0.104	0.89	—	—	0.99
TOPI 5RE ^a^	56	0.699	0.184	−0.65	0.104	0.92	—	—	0.94

^a^ The TOPI 5RE represents only 56 of the 66 items of the 5E that were carried forward from the TOPI 5 (Study 1) to the TOPI 5R (Study 2). The TOPI 5RE therefore lacks several more difficult items (i.e., high *b* in IRT terms), and we do not recommend the use of the 5RE for that reason.

## Footnotes

1 We have simplified our TOPI versioning system to render the names less cumbersome; see Section 1.3.2 for how to obtain more detail on this and other matters discussed here.

## Supplementary Materials

The Technical Supplement for this article is available online at: https://scholars.unh.edu/personality_lab/31.
